# Effect of ZnO on the Physical Properties and Optical Band Gap of Soda Lime Silicate Glass

**DOI:** 10.3390/ijms13067550

**Published:** 2012-06-18

**Authors:** Mohd Hafiz Mohd Zaid, Khamirul Amin Matori, Sidek Hj. Abdul Aziz, Azmi Zakaria, Mohd Sabri Mohd Ghazali

**Affiliations:** 1 Department of Physics, Faculty of Science, Universiti Putra Malaysia, UPM Serdang, Selangor 43400, Malaysia; E-Mails: mhmzaid@gmail.com (M.H.M.Z.); sidekaa@science.upm.edu.my (S.H.A.A.); Azmizak@gmail.com (A.Z.); mohdsabri@umt.edu.my (M.S.M.G.); 2 Advanced Materials and Nanotechnology Laboratory, Institute of Advanced Technology, Universiti Putra Malaysia, UPM Serdang, Selangor 43400, Malaysia; 3 Department of Science Physics, Faculty of Science and Technology, Universiti Malaysia Terengganu, Kuala Terengganu, Terengganu 21030, Malaysia

**Keywords:** glass, density, Ultra-Violet-Visible, optical band gaps

## Abstract

This manuscript reports on the physical properties and optical band gap of five samples of soda lime silicate (SLS) glass combined with zinc oxide (ZnO) that were prepared by a melting and quenching process. To understand the role of ZnO in this glass structure, the density, molar volume and optical band gaps were investigated. The density and absorption spectra in the Ultra-Violet-Visible (UV-Visible) region were recorded at room temperature. The results show that the densities of the glass samples increased as the ZnO weight percentage increased. The molar volume of the glasses shows the same trend as the density: the molar volume increased as the ZnO content increased. The optical band gaps were calculated from the absorption edge, and it was found that the optical band gap decreased from 3.20 to 2.32 eV as the ZnO concentration increased.

## 1. Introduction

Oxide glasses are among the few solids that transmit light in the visible region of the spectrum. There are various types of glass that have been studied by those involved in glass research. In the early period of glass research, most research focused on silica oxide as a glass-forming network. Among oxide glasses, phosphate and silicate glasses are the two most important materials, and they have been used extensively for lasers and fibre amplifiers [1,2]. Compared with silicate glasses, phosphate glasses are more limited in their use because they are hydroscopic in nature [3] and have a lower glass transition temperature. In contrast, silicate glasses exhibit superior chemical resistance and are optically transparent at the excitation and lasing wavelengths [4]. Therefore, they are more compatible with the fabrication process in the development of optical devices.

The study of optical absorption spectra provides a very productive method for investigating optically induced electronic transitions and an insight into the energy gap and band structure of crystalline and amorphous materials [5]. The principle of this technique is that a photon with an energy level greater than the band gap energy will be absorbed [6,7]. Absorption and transmission in the ultraviolet, visible and infrared regions are important in optical instruments. The absorption in all three regions can be used to study the short-range structure of glasses which encompasses the immediate surroundings of the absorbing atom [8].

The ability to control the physical properties of glasses, e.g., the refractive index and density, by variations in glass composition suggests the feasibility of chemically controlling the materials according to the needs of a given application [9]. Among the conventional glasses, soda-lime silicate (SLS) glasses have attracted much research attention because of their good glass-forming nature compared with several other conventional systems. Silicate glass is an attractive host matrix for transition metal ions because of its excellent optical and mechanical properties, good chemical stability, high UV transparency, low thermal expansion coefficient leading to strong thermal resistance, low non-linear refractive index, high surface damage threshold, large tensile fracture strength and good durability [10,11].

However, little information is available on the structure and properties of multi-component SLS glasses containing ZnO. A spirited debate exists in the literature concerning the effect of the addition of ZnO on the borate and tellurite glass system [12,13]; however, research on the topic of ZnO addition to the SLS glass system has been limited, and no definitive answers to the fundamental issues have been published. Although many properties of SLS glass, such as its desirable insulating properties, and mechanical parameters, have attracted the interest of a number of researchers because of the wide-ranging industrial and technical applications of SLS glass, no systematic study of the structural, elastic, and optical properties of SLS glass containing ZnO has been reported.

This research aims to study the structural, optical, elastic and physical properties of (ZnO)*_x_*(SLS)_1 −_ *_x_* glasses. The main obstacles when conducting this research are the difficulties in measuring the effect of ZnO addition on the structural, optical, and physical properties of SLS glass.

## 2. Results and Discussion

Table 1 shows the chemical composition of all samples and reveals the fact that increasing the ZnO content in the SLS glass resulted in the composition of SiO_2_ decreasing from 69.5 to 41.7 wt.%. The other major components in the glass samples, CaO and Na_2_O, also decrease with the addition of ZnO to SLS. CaO decreased from 11.3 to 6.8 wt.%, and Na_2_O decreased from 12.5 to 7.5 wt.% as the ZnO content increased. Other minor components in the (ZnO)*_x_*(SLS)_1 −_ *_x_* glasses also decreased as the ZnO content increased; Al_2_O_3_ decreased from 2.8 to 1.6 wt.%, K_2_O decreased from 1.5 to 0.9, MgO decreased from 2.0 to 1.3 wt.% and the composition of Fe_2_O_3_, B_2_O_3_ and BaO was 0.1 wt.% in the (ZnO)*_x_*(SLS)_1 −_ *_x_* glasses.

**Figure 1:**
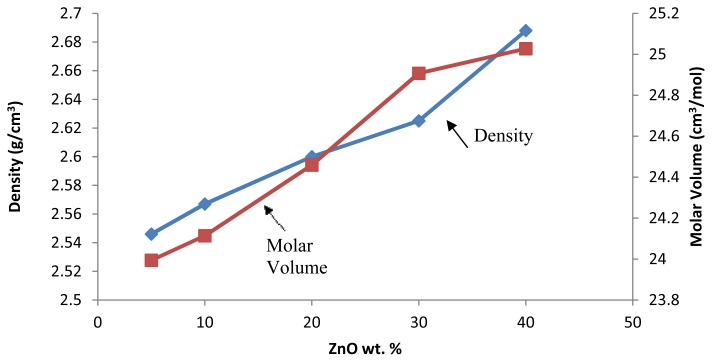
The density (left axis) and molar volume (right axis) of (ZnO)*_x_*(SLS)_1 −_ *_x_* glasses.

A plot of the density and molar volume of the glasses *versus* the weight percentage of ZnO is shown in Figure 1. Figure 1 shows that the density of the (ZnO)*_x_*(SLS)_1 −_ *_x_* glass was increased with the addition of ZnO content. The increased density of the glasses is due to the heavier zinc atomic weight compared with the other elements in the glass samples. The atomic weight of zinc is 65.390, which is heavier than the atomic mass of Si (28.086), Ca (40.078) and Na (22.989). Increases in the density of the (ZnO)*_x_*(SLS)_1 −_ *_x_* glass sample also result in changes in the crosslink density. The increases in the density of the glass samples are attributed to the formation of new linkages in the (ZnO)*_x_*(SLS)_1 −_ *_x_* glass structure. The Zn^2+^ ion tends to occupy interstitial sites within the highly open glass network.

The addition of ZnO to the SLS glass network causes structural rearrangement of the atoms [14]. As reported earlier, the structure of the SLS glass network consists of the following structural units: SiO_4_ trigonal bipyramids and SiO_3_ trigonal pyramids [15–17]. There is 4-coordination of Si in the tetragonal form, with the nearest-neighbours being arranged at four of the vertices of a trigonal bipyramid, suggesting the considerable covalent character of the Si–O bonds. An oxygen atom introduced into glass with a high SiO_2_ content opens a Si–O–Si bridge and changes two SiO_4_ units into SiO_3_ units. The increase in the density is due to glass structural changes caused by the influence of Zn^2+^ in breaking the Si–O networks. All oxygen atoms from ZnO are used to rupture the Si–O–Si bridges, which is accompanied by the transformation of nearly all participating SiO_4_ groups into SiO_3_ groups [16,17].

The molar volume of the glasses increased when the ZnO content increased. The values of *V*_m_ increased from 23.927 to 25.028 cm^3^·mol^−1^. The increase in the *V*_m_ indicates an increase in the inter-atomic distance, and ZnO acts as a modifier. In this case, the non-bridging oxygens (NBOs) are increased in number in the SLS network; therefore, *V*_m_ increases. Therefore, the compactness of the glass sample will decrease.

**Figure 2:**
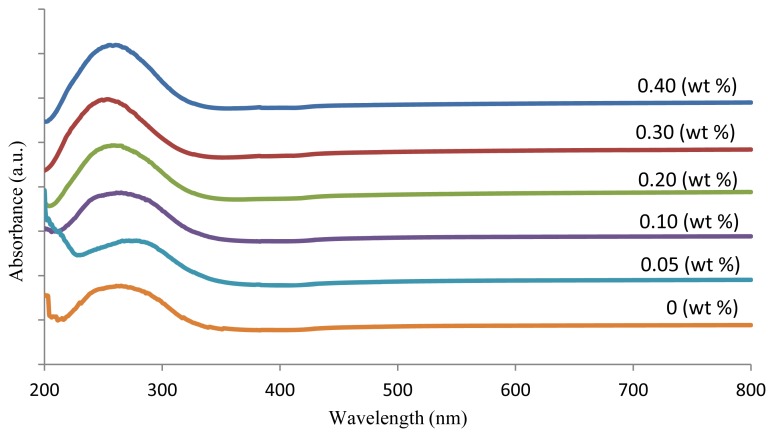
Optical absorption spectra of (ZnO)*_x_*(SLS)_1 −_ *_x_* glasses.

Absorption spectra of the SLS glasses containing ZnO are shown in Figure 2. It is clear that there is no sharp absorption edge that corresponds to the characteristics of the glassy state. Generally, the absorption edge of the glass samples is determined by the strength of the oxygen bonding in the glass network.

The changes in oxygen bonding in the glass network (the formation of non-bridging oxygens, NBOs) will change the characteristics of the absorption edge. As the material absorbs a photon of incident light, an electron is excited to a higher energy level. This transition of the electron can be direct (without a phonon-assisted mechanism) or indirect.

In the direct transition, an electron produced by the energy difference as a photon of light is transferred from the conduction band to the empty state in the valence band. For the indirect transition, an electron in the conduction band is indirectly transferred to the valence band and undergoes a momentum change and a change in energy. This difference between the direct and indirect band structure is very important for choosing which material can be used in a device according to the light energy requirements.

**Figure 3:**
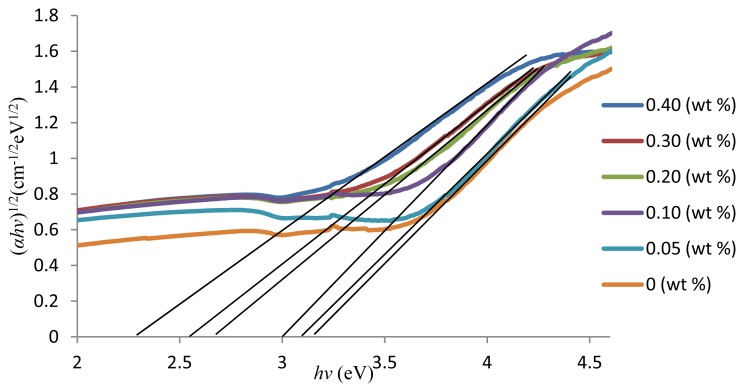
The (*αhv*)^1/2^ as a function of photon energy, *hv* for (ZnO)*_x_*(SLS)_1 −_ *_x_* glasses.

In this work, the absorption coefficients, *A*, was determined near the absorption edge of different photon energies for all glass samples using Equation (4). Therefore, the typical plot of (*Ahv*)^1/2^ *versus* photon energy (*hv*) for indirect allowed transitions that is used to determine the values of optical band gaps, *E*_opt_, is shown in Figure 3. There is a linear dependence between (*Ahv*)^1/2^ and the photon energy. At the higher photon energies, the transitions occurring in the present glass samples were indirect. The indirect energy band gap is determined from the linear region of the plot and is shown in Figure 3. The results show that the indirect band gap values decrease from 3.20 to 2.32 eV with the increase in the ZnO content. These results suggest that the covalent nature of the glass matrix decreases with the increase in the ZnO content.

In the glass sample, ZnO is treated as a glass modifier. The addition of alkali oxides, alkaline earth oxide and many other divalent metal oxides causes depolymerisation of the glass chain [18]. Consequently, the average oxygen bond chain length is shortened. The addition of oxides also opens up the chain by breaking those oxygen bonds. In this way, the amount of non-bridging oxygen (NBO) grows as the ZnO concentration increases, as described by the mechanism. This process changes the oxygen bonding in the glass-forming network. Any changes in the oxygen bonding in a glass network, such as the formation of NBOs, will results in changes in the absorption characteristics. This alteration explains the decrease in the optical band gap with an increase in the ZnO content.

**Figure 4:**
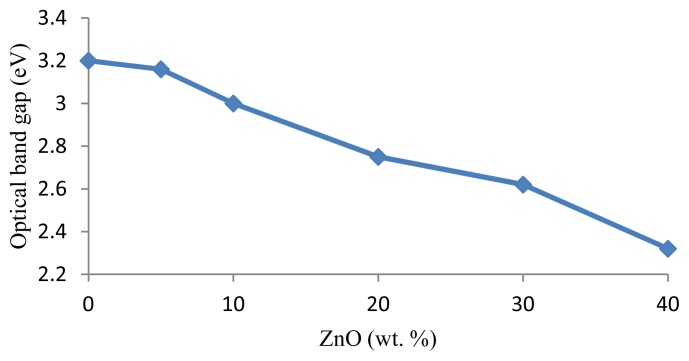
The optical band gap (ZnO)*_x_*(SLS)_1 −_ *_x_* glasses.

Figure 4 shows the variation of *E*_opt_ for different weight percentages of ZnO. The values of *E*_opt_ decrease linearly with increasing ZnO content. Such variation can be explained by the proposition that the NBO content increases as the ZnO content increases, shifting the band edge to higher energies and leading to a decrease in the value of *E*_opt_.

## 3. Experimental Section

Five series of glass samples were prepared by mixing together specified weights of ZnO (99.99%, Aldrich) and SLS glass powder (waste glass bottle). To produce the SLS powder, the waste SLS glass bottle was crushed using a mortar and pestle. The SLS powder was ground to a size of <200 μm. The SLS powder and ZnO powder were weighed using an electronic digital balance to prepare the series of glass samples. The accuracy of the electronic digital weighing was ±0.001 g. The SLS powder and ZnO powder were weighed to obtain ~100 g of mixture and then mixed thoroughly.

After the mixing process, the mixture was transferred to a milling container containing milling balls of different sizes. The dry milling process is a good technique to produce fine glass powders. After that, the milling container was transferred to the milling machine, and the mixture was milled for 18 h. When choosing the glass melting procedures to use, the first criterion that needed to be considered was the melting temperature. Inorganic glasses are commonly melted at least 100 °C above the liquidus temperature to achieve a viscosity that is low enough to allow homogenisation and refinement to take place. This melting temperature depends on the oxide composition of the materials. In this study, the temperature used for the melting process was 1300 °C.

The batches of mixture were transferred to an alumina crucible and preheated at 400 °C for a period of 1 h to reduce the volatilization tendency. After that, the crucible was transferred to an electric furnace at 1300 °C for 2 h to ensure completion of homogeneous melting. After that, the stainless steel mould should be free from dust and impurities and can be preheated at 400 °C for 30 min. The process of pouring the melt into the stainless steel mould should be performed quickly to prevent the solidification process from occurring at the same time.

The melt solidified rapidly at room temperature, and the glass sample was then annealed in the electrical furnace for 1 h at 400 °C, which is below the glass transition temperature. The annealing process was performed to the glass sample to stabilise the glass structure and to reduce the tensile and thermal stress of the glass sample. The furnace was then switched off, and the glass samples were allowed to cool down to room temperature.

An X-ray diffraction (XRD) investigation was made with a Philips X-ray diffractometer with Cu-Kα radiation in the 2θ range from 10° to 90° using 0.02° steps. The amorphous nature of these glass samples was established and confirmed using X-ray diffraction (XRD; PANalytical X’pert PRO PW 3040 MPD X-ray powder diffractometer). The glasses that were successfully prepared were transparent and free of bubbles.

The fluorescence X-ray spectrometer EDX-720/800HS/900HS was used to determine the chemical compositions of the glass samples. The X-rays irradiate the surface of the sample, and the fluorescence X-rays in the analyser detect the elements in the sample. The data obtained from the analyser is sent to the workstation of the EDXRF.

The density (*ρ*) of the prepared glass samples was measured at room temperature by the standard Archimedes principle (apparent weight loss) using acetone as the immersion fluid. The sample was first weighed in air, W_air_, and then in an immersion liquid (acetone), W_ac_, with the following density: *ρ*_ac_ = 0.789 g·cm^−3^. The weighing process was performed with an electronic balance. The density of the sample was then calculated using the following relationship (Equation (1)):

(1) $$\rho ={\text{W}}_{\text{air}}\rho_{\text{ac}}/({\text{W}}_{\text{air}}-{\text{W}}_{\text{ac}})$$

where the estimated error was ±0.001 g·cm^−3^.

The molar volume (*V*_m_) was measured in cubic centimetres per mole (cm^3^ mol^−1^) for liquids and solids and can be expressed as (Equation (2)):

(2) $$V_{\text{m}}=\sum M_{\text{T}}/\rho$$

where *M*_T_ is the total molecular weight of the multi-component glass system given as (Equation (3)):

(3) $$M_{\text{T}}=x_{i}Z_{i}$$

where *x_i_* is the mole fraction of the *i*^th^ oxides, and *Z_i_* is the molecular weight of the *i*^th^ oxides.

A UV-Vis Spectrophotometer (Lambda 35, Perkin Elmer) was used to measure the optical band gap energy of the glass samples series. The transmission signal was measured for the wavelength from 200 to 800 nm and then converted to an absorption signal for further evaluation [19]. The measurement of the absorption spectrum in glass leads to determination of the optical band-gap energy. Commonly, UV-Vis spectroscopy is carried out by dispersing the powdered samples in solutions, such as deionised water, acetone, ethanol or other alcohols.

One of the major problems with this approach is that samples often precipitate due to the particle size not being small enough, making the absorption spectrum difficult to analyse. To avoid these difficulties, it is preferable to use a Reflectance Spectroscopy Accessory (RSA), which reliably obtains the optical band gap of powder samples. Morales *et al.* [20] used diffuse reflectance spectroscopy for optical property measurements of powdered nanostructures. It was assumed that for this study, the fundamental absorption edge of the glass is due to the indirect transition. The optical band gap energy is given by Equation (4) [21]:

(4) $${\left(Ahv\right)}^{1/2}=C(hv-E_{\text{opt}})$$

near the optical band gap, where *A* is the optical absorption coefficient, *C* is a constant that is independent of the photon energy (*hv*) and *E*_opt_ is the indirect allowed optical band gap energy. From the plot of (*Ahv*)^1/2^ against *hv*, the value of the optical band gap energy, *E*_opt_, is obtained by extrapolating the linear fitted regions to (*Ahv*)^1/2^ = 0.

## 4. Conclusions

In this study, a series of glass samples was successfully prepared using melting and quenching techniques. From the XRD measurements, the glass samples showed a broad halo characteristic, which reflected the characteristics of the amorphous or glass structure. The chemical compositions of the glass samples were successfully determined using the EDXRF technique. The density and the molar volume of the (ZnO)*_x_*(SLS)_1 −_ *_x_* glasses increased as the ZnO content increased. The increase in the density of the glass is due to the heavier zinc atomic weight compared with the other elements in the glass samples. The increase in the molar volume of the glass is due to the glass structural changes. A UV-Vis spectrophotometer (Lambda 35, Perkin Elmer) was used to measure the optical band gap energy of a series of glass samples. The results obtained show that the optical band gap decreased as the ZnO content increased.

## Figures and Tables

**Table 1:** Analysis of the chemical composition of (ZnO)*_x_*(SLS)_1−_*_x_* glasses using EDXRF.

Sample: *x*	SiO_2_	CaO	Na_2_O	Al_2_O_3_	K_2_O	MgO	Fe_2_O_3_	B_2_O_3_	BaO	ZnO
0	69.5	11.3	12.5	2.8	1.5	2.0	0.2	0.1	0.1	0
0.05	66.0	10.7	11.9	2.6	1.4	2.0	0.1	0.1	0.1	5.1
0.10	62.6	10.2	11.3	2.4	1.3	1.9	0.1	0.1	0.1	10.0
0.20	55.6	9.1	10.0	2.2	1.2	1.7	0.1	0.1	0.1	19.9
0.30	48.7	7.9	8.8	1.9	1.1	1.5	0.1	0.1	0.1	29.8
0.40	41.7	6.8	7.5	1.6	0.9	1.3	0.1	0.1	0.1	39.9
